# Genetic chimerism of *Vitis vinifera *cv. Chardonnay 96 is maintained through organogenesis but not somatic embryogenesis

**DOI:** 10.1186/1471-2229-5-20

**Published:** 2005-09-29

**Authors:** Christophe Bertsch, Flore Kieffer, Pascale Maillot, Sibylle Farine, Gisèle Butterlin, Didier Merdinoglu, Bernard Walter

**Affiliations:** 1 Université de Haute-Alsace, Laboratoire Vigne Biotechnologies et Environnement, 33, rue de Herrlisheim 68000 Colmar, France; 2 INRA, UMR 1131, Vigne et Vin d'Alsace, 28, rue de Herrlisheim 68000 Colmar, France

## Abstract

**Background:**

Grapevine can be a periclinal chimera plant which is composed at least of two distinct cell layers (L1, L2). When the cell layers of this plant are separated by passage through somatic embryogenesis, regenerated plants could show distinct DNA profiles and a novel phenotype which proved different from that of the parent plant.

**Results:**

Genetically Chardonnay clone 96 is a periclinal chimera plant in which is L1 and L2 cell layers are distinct. Plants obtained via organogenesis through meristematic bulks are shown to be composed of both cell layers. However, plants regenerated through somatic embryogenesis starting from anthers or nodal explants are composed only of L1 cells. These somaclones do not show phenotypic differences to the parental clone up to three years after regeneration. Interestingly, the only somaclone showing an atypical phenotype (asymmetric leave) shows a genotypic modification.

**Conclusion:**

These results suggest that the phenotype of Chardonnay 96 does not result from an interaction between the two distinct cell layers L1 and L2. If phenotype conformity is further confirmed, somatic embryogenesis will result in true-to-type somaclones of Chardonnay 96 and would be well suitable for gene transfer.

## Background

Improvement of grapevine rootstocks and scion varieties can be achieved through either inter-specific hybridization or clonal selection. Grapevine clonal selection consists in choosing in a variety one plant presenting desired characteristics. This selected plant is further propagated by vegetative multiplication known to maintain trueness-to-type. But clonal selection is restricted to the natural variability of a given cultivar, within the limits of the characteristics on which the trueness-to-type is based. Somatic embryogenesis could be an additional possibility for varietal improvement. A broader perspective of improvement of grapevine cultivars for characteristics such as resistance to pests and diseases has been opened by transgenic technologies which in most cases are based on somatic embryogenesis of grapevine [1] and shoot organogenesis [2]. One question to be addressed is the phenotypic and genotypic variability of grapevine clones raised through somatic embryogenesis or shoot organogenesis. Recently, Desperriers *et al*. [3] presented results of a ten years observation of *Vitis vinifera *Gamay somaclones which showed variations in fertility as well as sugar content, size and level of maturity of the grapes. These observations illustrate that somaclones can differ from the original parent without changing the fundamental typicity of the wine. One possible origin of variability may be the separation of cell layers of the mother plant from which clones are grown.

The grapevine meristem is considered to be composed at least of two distinct cell layers L1 and L2 [4], which can produce a chimeric tissue structure. For example, *Vitis vinifera *cv. Pinot Meunier phenotype is due to the interaction of genetically distinct cell layers. When the cell layers of Pinot Meunier periclinal chimera were separated by passage through somatic embryogenesis, regenerated plants showed distinct DNA profiles which proved to be different from that of the parent plant. Regenerated somaclones also showed a novel phenotype [5].

About five hundred microsatellite markers of the grapevine genome are now available and widely as well as very efficiently used for identification of cultivars [6-8]. Riaz *et al. *[9] used microsatellite markers for the detection of reproducible intra-cultivar polymorphism in *Vitis vinifera *Chardonnay and Pinot noir. Furthermore, some of these microsatellite markers made it possible to differentiate the two cell layers L1 and L2 in some clones of both cultivars [9,10].

A previous paper [11], showed that in the leaf tissue of Chardonnay 96 the microsatellite marker VMC 5g7 revealed two standard alleles (198:220 bp) and a variant allele (222 bp) previously defined by Riaz *et al*. [9]. Wood tissues and roots only presented the two standard alleles with VMC 5g7. With a second microsatellite marker (VMC 6g10), two standard alleles (114:140 bp) and a mutant allele (142 bp) were detected in leaves. This mutant allele replaced one of the standard alleles in woods and roots (114:142 bp). In addition, we showed, that somaclones regenerated from anthers of a single inflorescence all derived from L1 cells exclusively.

In the present paper we report results of a wider genotypic analysis of somaclones regenerated either from anthers of different inflorescences or from nodal explants of Chardonnay 96. The same microsatellite markers were also used to compare the genotype of different clones obtained through shoot organogenesis. The genotypic identities (L1; L2) of the regenerated clones are in accordance with their respective phenotypic characteristics.

## Results

### Genotypic analysis of various tissues of Chardonnay 96 mother clone

Three alleles were detected in leaves of Chardonnay 96 mother clone with the microsatellite marker VMC 6c10: the 114 bp and 140 bp standard alleles and an additional 142 bp variant allele (table 1). The standard alleles were defined by Riaz *et al. *[9] as the most frequently detected alleles in different clones of the same cultivar. VMC 5g7 revealed in leaves the two 198 bp and 220 bp standard alleles and a 222 bp variant allele (table 1). The same genotype was observed with leaves from *in vitro *or greenhouse-grown plants. In the DNA extracted from rootlets and wood tissues, only the two pairs of alleles 114:142 and 198:220 were detected with VMC 6c10 and VMC 5g7 respectively (table 1). In berry skin, the triallelic profiles were detected with both markers. It is known that wood and roots are composed of L2 cells only and that leaves comprise L1 and L2 cell layers. Our results lead to the conclusion that the VMC 6c10 114 bp and 142 bp alleles as well as the VMC 5g7 198 bp and 220 bp alleles are present in L2 cells of Chardonnay 96. We can also deduce that L1 cells have the VMC 6c10 114 bp and 140 bp alleles and the VMC 5g7 the 198 bp and 222 bp alleles.

### Regeneration of plants through somatic embryogenesis or shoot organogenesis

**Figure 1:**
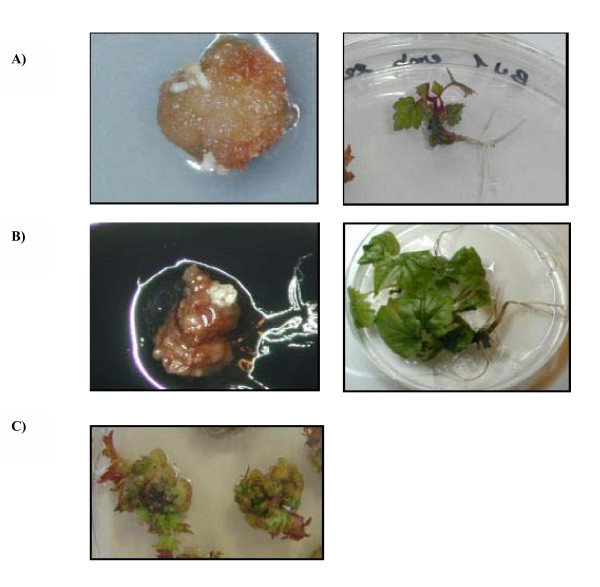
**Somatic embryogenesis and shoot organogenesis of *Vitis vinifera *Chardonnay 96. **A) Embryogenic callus from anthers and plant regeneration, B) Embryogenic callus from nodal explants and plant regeneration, C) Shoot organogenesis from meristematic bulk.

Four different inflorescences of Chardonnay 96 were used to collect anthers. Primary somatic embryos were obtained from anther-derived embryogenic calli after a 2 month period (figure 1A). Secondary embryos were obtained 1 month after initiation of embryogenic calli from primary embryos. Embryogenic calli and primary embryos from nodal explants were obtained after 2–4 months (figure 1B). Meristematic bulks developed after successive transfers of shoots on IM medium with benzyladenine concentrations increasing up to 13.2 μM. High numbers of adventitious shoots regenerated from slices cut from the meristematic bulk tissues, and rooted plantlets further developed (figure 1C).

Efficient plant growth was further obtained with a number of somaclones. The plants did not show any atypical phenotype in visual comparison with vegetatively propagated Chardonnay 96 grown in the growth chamber and the greenhouse. Except one somaclone (n° 21) originated from an anther-derived callus.

### Genotypic analysis of Chardonnay 96 somaclones obtained from anthers and from nodal explants

The same genotypic profiles were obtained with a total of 29 primary and 7 secondary somaclones regenerated from anthers collected from 4 different inflorescences and 16 clones from nodal explants (table 2). In leaves from *in vitro *and greenhouse-grown plants, the standard genotype (114:140) was visualised with VMC 6c10. For VMC 5g7 in addition to the standard 198 bp allele, the 222 bp variant allele was detected in leaves from all somaclones (table 2). In the rootlets, the standard diallelic genotype was visualised with VMC 6c10. With VMC 5g7 the 198 bp standard allele and the 222 bp variant allele were detected (table 2). These results show that all somaclones regenerated from L1 cells of the Chardonnay 96 explants.

### Phenotypic and genotypic analysis of somaclone n°21

**Figure 2:**
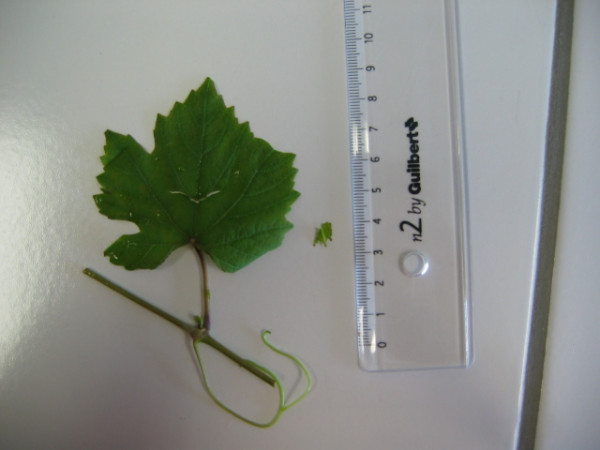
Asymmetric leaf of a 3 year-old atypical somaclone n°21 plant.

Compared to the Chardonnay 96 mother clone, which presents mature circular leaves undivided or with five lobes, all leaves from somaclone n° 21 show one circular undivided half and the other half with separated lobes (figure 2). This phenotype was observed after the acclimatization and is still observed after 3 years on greenhouse-grown plants only for this somaclone.

In leaves from *in vitro *and greenhouse-grown somaclone 21, the triallelic profile was detected with VMC 6c10. For VMC 5g7 in addition to the standard 198 bp allele, the 222 bp variant allele was detected. The same results were obtained with berry skin. In the rootlets, the standard allele (114) and the mutant allele (142) were visualised with VMC 6c10 and with VMC 5g7 the standard allele (198) and the mutant allele (222) were detected (table 3). These results indicate that the genotype of somaclone 21 is the same as that of the mother clone for VMC 6c10, but for VMC 5g7 only 2 alleles present in the L1 cell layer of the mother plant are present. A mutation in the microsatellite sequence VMC 6c10 from the L1 cell type of the mother clone probably occurred during embryogenesis of clone 21.

### Genotypic analysis of clones obtained through shoot organogenesis

Leaves from all 7 clones obtained through shoot organogenesis show the triallelic profile both for VMC 6c10 and VMC 5g7 (table 2). This suggests that plants obtained via organogenesis through meristematic bulks are derived from both L1 and L2 cell layers of the Chardonnay 96 tissues from which they grew.

## Discussion

### *Vitis vinifera *Chardonnay clone 96 is a periclinal chimera

In grapevine, apical meristems are composed of two or more cell layers forming the tunica in addition to a corpus [4,12]. In the leaf tissue from Chardonnay 96, which is derived from both the outer tunica layer L1 and the inner cell layer L2, the microsatellite marker VMC 5g7 revealed the two standard alleles and a variant allele previously defined by Riaz *et al. *[9]. Wood tissues and roots, which originate exclusively from the L2 layer, presented only the two standard alleles. The presence of a third allele in leaf suggests that Chardonnay is a periclinal chimera in which a mutant allele is present only in the L1 layer, as described by Riaz et al. [9]. For the microsatellite marker VMC 6g10, a mutant allele was detected in leaves, wood tissue and roots. This mutant allele replaced one of the standard alleles in woods and roots, whereas in leaves the mutant allele was present simultaneously with the two standard alleles [11]. These results suggest that the 2 bp-mutation resulting in the replacement of the VMC 6c10 140 bp allele by the 142 bp allele only occurred in L2. Riaz *et al. *[9] propose that the mutation most likely occurred in an L1 or L2 cell and then came to populate both layers of the meristem rather than two identical mutations appearing independently in the L1 and L2. Results reported by Riaz *et al*. [9] were based on visual interpretation of electrophoretic profiles of amplification products. We suggest that such observations could be biased by the DNA polymerase slippage. Our analyses with ABI PRISM allowed to differentiate between an allele and the different stutter bands, without ambiguity.

### Somaclones develope only from L1 cells of Chardonnay 96

In the present study, we show that somaclones obtained not only from anthers from different inflorescences but also from nodal explants all derived from L1 cells. Embryogenic calli were composed exclusively of cells showing the genetic profile of L1 cells of the mother clone Chardonnay 96 (data not shown), suggesting that L2 cells could not multiply into callus at least in our culture conditions. On another hand, clones raised through shoot organogenesis are composed of cells showing the genetic profiles of both L1 and L2 cells of the mother clone. These observations suggest that the genetic chimerism of Chardonnay 96 is maintained through shoot organogenesis but not through somatic embryogenesis.

Anthers are the most widely used organ for initiation of grapevine somatic embryos. It has been shown that embryogenic cells derive from the anther filament. Both L1 and L2 cell layers seem to be competent to form embryogenic calli in some conditions, as reported for Pinot Meunier by Franks *et al. *[9]. Filaments from anthers of Chardonnay 96 are composed of L1 and L2 cells (data not shown). But, in our conditions only L1 cells of Chardonnay 96 developed into embryos. A similar result was reported recently with Pinot gris from which only the L1 cell layer is competent to form embryogenic callus [10].

### Phenotype variation of Chardonnay 96 somaclones

Embryogenesis might generate new grapevine phenotypes when the mother plant is a chimera of genetically distinct cell layers. For example, the separation of chimeric cell layers of Pinot Meunier through somatic embryogenesis generated plants that had distinct DNA profiles and had novel phenotypes which were different from those of the parent plants [5]. The phenotype of all the somaclones we obtained from anthers has been observed for 2–3 years in a glasshouse: no visible modification was noticed (except for the somaclone n°21) in comparison to the mother clone Chardonnay 96. This phenotypic conformity suggests that the L2 genotype would not significantly participate in the phenotypic expression in the L1–L2 chimeric Chardonnay 96. More subtle variations in fruit setting and wine quality may be only detected in the future, when the somaclones will be grown in the vineyard. Variation of colour intensity, sugar content, size and level of maturity of the grapes has been reported for adult somaclones of Gamay after ten years observation [3]. Though no genotypic analyses were done for the different Gamay somaclones, it can be hypothesised that this somaclonal variation is of epigenetic origin [13]. The Chardonnay 96 mother clone shows the typical ampelographic traits of the cultivar Chardonnay with mature circular leaves undivided or with five lobes, petiolar sinus slightly open, often limited through veins at petiole end. Some adult leaves of somaclone 21 have an asymmetric shape, one half undivided or slightly lobed, the other half deeply lobed. Somaclone 21 probably arose from L1 cell(s) of Chardonnay 96 as all the other somaclones. We suggest that during an early cell division a mutation occurred in locus 6c10 which replaced the 140 bp allele by a 142 bp allele. The L2 cell layer of somaclone 21 originated from the diallelic mutated cell (114:142), whereas cells with the non mutated diallele (114:140) further multiplied giving the L1 cell layer of somaclone 21. This hypothesis is in accordance with the fact that the most frequently observed allelic size variation is the addition of one motif [14].

## Conclusion

Regeneration via embryogenesis for Chardonnay 96 could result generally in a non chimeric plant and in an unchanged phenotype and would be well suited for gene transfer.

## Methods

### Plant material

*Vitis vinifera *cv. Chardonnay clone 96, was obtained from ENTAV (Etablissement National Technique pour l'Amélioration de la Viticulture, Le Grau du Roi, France), the national repository for registered grape clones in France. Forced adult plants were maintained in a growth chamber at 25 ± 0.5°C, 70 ± 10 % RH with a 16 h – photoperiod.

### Initiation of embryogenic calli from anthers

Anthers were dissected and grown as described by Mauro *et al. *[15]. For long-term culture of embryogenic callus, subcultures were performed every three weeks on the MPM medium described by Perrin *et al. *[16]. All the cultures were maintained at 25 ± 0.5°C, 70 ± 10 % RH with a 16 h – photoperiod, except the very first step -the culture of detached anthers- which was performed in the dark.

For production of secondary embryos, primary embryos were cut off and transferred onto half strength Murashige and Skoog medium (MS medium) [17] supplemented with 20 g.l^-1 ^sucrose, 0.7% Bacto-agar, 2.5 μM 2,4-dichlorophenoxyacetic acid (2,4-D) and 0.5 μM 6-benzylaminopurine (BA; N6-benzyladenine). Incubation was done at 25 ± 0.5°C in the dark, during 3 weeks. Then calli were subcultured every three weeks on MPM medium and maintained under the same conditions as previously described.

### Initiation of embryogenic calli from nodal explants

Nodal explants were excised from plantlets grown *in vitro *and plated on half strength MS medium supplemented with 25 g.l^-1 ^sucrose, 0.7 % Bacto-agar, 9 μM 2,4-D and 4.5 μM BA. Subculture was performed on half strength MS medium supplemented with 60 g.l^-1 ^sucrose, 0.7 % Bacto-agar, 20 μM indole-3-acetic acid (IAA), 10 μM 2-naphtoxyacetic acid (NOA) and 1 μM BA. Embryogenic calli were obtained after several transfers onto this last medium. For long-term culture of embryogenic callus, subcultures were performed every three weeks on the same medium. All the cultures were maintained at 25 ± 0.5°C, 70 ± 10 % RH with a 16 h – photoperiod, except the callus initiation which was performed in the dark.

### Regeneration and acclimatization of somaclones

Embryos were carefully excised and transferred onto half-strength MS medium containing 20 g.l^-1 ^sucrose, 0.7 % agar and 0.4 μM BA. After two weeks at 25 ± 0.5°C and 16 h light, the growing embryos were individually transferred into tubes containing half-strength MS medium without plant growth factor. Plantlets with rootlets were transferred to soil and allowed to acclimatize in a growth chamber for about three weeks before transfer to a greenhouse.

### Ampelographic observation

Conformity of the different 3 years old somaclones were based on visual observation of leaf morphology and phyllotaxy compared to Chardonnay clone 96.

### Shoot organogenesis

*In vitro *propagation of *V. vinifera *Chardonnay 96 was initiated on MS basal medium supplemented with 4.4 μM BA, 30 g.l^-1 ^sucrose and 0.7 % Bacto agar. Meristematic bulks were initiated on IM medium of Mezzetti *et al. *[2] supplemented with 0.05 μM NAA (α-naphtalene-acetic acid) and increasing concentrations of BA : 4.4 μM for the first 30 days subculture, 8.8 μM for the second subculture and finally 13.2 μM. At each transplantation, the apical dome was eliminated.

In order to induce shoot organogenesis, thin slices were cut from the inner part of the meristematic bulks and transferred onto IM medium containing 13.2 μM BA. Growing shoots were transferred to rooting medium of Quoirin and Lepoivre [18] containing 4.9 μM indole-3-butyric acid (IBA) and 5.7 μM IAA [2]. Growing plantlets were further propagated on hormone free MS medium with half strength macroelements.

### DNA extraction

Leaves were harvested from plants regenerated through somatic embryogenesis or shoot organogenesis: these plants were *in vitro *and greenhouse-grown somaclones and *in vitro*-grown clones from organogenesis. Leaves from greenhouse-grown plants of Chardonnay 96 mother clone were used as a control. Rootlets were taken from *in vitro-*grown plantlets. Wood tissue was obtained from the dormant canes after the bark and cambium were first scraped away. Berry skin, removed using a scalpel, was also analysed. About 80 mg tissue was ground in liquid nitrogen using a grinder (Retsch MM200) and total DNA was extracted with the Qiagen Dneasy Plant mini-kit (Qiagen, Hilden, Germany) as described by the supplier.

### Amplification of microsatellites and polymorphism detection

DNA was analysed using two pairs of primers flanking two different microsatellite regions: VMC6c10 and VMC 5g7 (Vitis Microsatellites Consortium, Dr. Rosa Arroyo Garcia and Dr. Kirsten Wolff), respectively marked with the fluorophores HEX and FAM. Amplification reactions were performed in a total volume of 50 μl with 10 ng of template DNA, 0.35 μM of forward primer labelled either with 6-FAM or HEX fluorophore, 0.35 μM of non-labelled reverse primer, 200 μM dNTP (Invitrogen), 1.5 mM MgCl_2_, 1X PCR Buffer and 0.2 unit Platinium^® ^Taq DNA Polymerase (Invitrogen). The PCR was carried out using a GeneAmp^® ^PCR System 2700 thermocycler (Applied Biosystems). The cycling program consisted of the following steps: 2 min at 94°C followed by 35 cycles of 40 s at 92°C, 1 min at 57°C and 1 min at 72°C and a final extension step of 7 min at 72°C. The amplification products were separated by capillary electrophoresis and detected with an ABI PRISM 310 Genetic Analyser (Applied Biosystem), using HD400-ROX as an internal size standard. The PCR fragments were detected with the GeneScan™ analysis software version 3.1 and the alleles were scored using the Genotyper™ DNA fragment analysis software version 2.5.2 (Applied Biosystems).

## Authors' contributions

CB carried out the molecular studies, participated in the analyses of tissues and extract, and drafted the manuscript. FK and SF participated in the production of embryogenic calli from anthers. PM participated in the production of embryogenic calli from nodal explants. GM and DM partipated in the analyses of tissues. BW initiated shoot organogenesis and envisioned and supervised all the studies, as well as drafting the manuscript.

## Figures and Tables

**Table 1:** Genotypes of Chardonnay clone 96 at the VMC 5g7 and VMC 6c10 loci.

Locus alleles (bp)			
DNA origin	VMC 6c10	VMC 5g7	cell layer

Leaves	114:140:142	198:220:222	L1 + L2
Wood	114:142	198:220	L2
Rootlets	114:142	198:220	L2
Berry skin	114:140:142	198:220:222	L1 + L2

**Table 2:** Genotypes at the VMC 5g7 and VMC 6c10 loci of clones regenerated through embryogenesisor shoot organogenesis

	Locus alleles (bp)			
	
Plant origin	DNA origin	VMC 6c10	VMC 5g7	cell layer of origin
***anthers***				
*Inflorescence A*				
Primary (20) or secondary (6) somaclones	Leaves *iv + gh*	114:140	198:222	L1
	Rootlets *iv*	114:140	198:222	L1
				
**Inflorescence B**				
Primary somaclones (3)	Leaves *iv*	114:140	198:222	L1
				
*Inflorescence C*				
Primary (5) or secondary (1) somaclones	Leaves *iv + gh*	114:140	198:222	L1
				
**Inflorescence D**				
Primary somaclone (1)	Leaves *iv + gh*	114:140	198:222	L1
				
***nodal explants***				
**Primary somaclones **(16)				
	Leaves *iv*	114:140	198:222	L1
	Rootlets *iv*	114:140	198:222	L1
				
***meristematic bulk***				
**clones **(7)				
	Leaves *iv*	114:140:142	198:220:222	L1 + L2

from:

– anthers from 4 different inflorescences (A, B, C, D)

– nodal explants

or through shoot organogenesis from meristematic bulks

(N°) : number of clones, iv : *in vitro*, gh : greenhouse

**Table 3:** Genotype of the somaclone 21 at the VMC 5g7 and VMC 6c10 loci

Locus alleles (bp)		
DNA origin	VMC 6c10	VMC 5g7

Leaves	114:140:142	198:222
Rootlets	114:142	198:222
Berry skin	114:140:142	198:222
